# Computational network models for forecasting and control of mental health trajectories in digital applications

**DOI:** 10.1038/s41746-025-02252-3

**Published:** 2025-12-30

**Authors:** Janik Fechtelpeter, Christian Rauschenberg, Christian Goetzl, Selina Hiller, Eva Wierzba, Niklas Emonds, Silvia Krumm, Ulrich Reininghaus, Daniel Durstewitz, Georgia Koppe

**Affiliations:** 1 Hector Institute for Artificial Intelligence in Psychiatry, Central Institute of Mental Health (CIMH), Medical Faculty Mannheim, Heidelberg University (UHD), Heidelberg, Germany; 2 Dept. of Psychiatry and Psychotherapy, CIMH, Medical Faculty Mannheim, UHD, Heidelberg, Germany; 3 Dept. of Theoretical Neuroscience, CIMH, Medical Faculty Mannheim, UHD, Heidelberg, Germany; 4 RG Machine Learning Human Behavior, Interdisciplinary Center for Scientific Computing, UHD, Heidelberg, Germany; 5 Dept. of Public Mental Health, CIMH, Medical Faculty Mannheim, UHD, Heidelberg, Germany; 6 German Center for Mental Health (DZPG), partner site Mannheim-Heidelberg-Ulm, Mannheim, Germany; 7 Department of Forensic Psychiatry and Psychotherapy, Ulm University, Ulm, Germany; 8 Technical University of Munich, TUM School of Medicine and Health, Department of Psychiatry and Psychotherapy, TUM University Hospital, Munich, Germany; 9 Department of Psychiatry and Psychotherapy, Medical Faculty, Leipzig University, Leipzig, Germany; 10 Health Service and Population Research Dept., Institute of Psychiatry, Psychology & Neuroscience, King’s College London, London, UK; 11 ESRC Centre for Society and Mental Health, King’s College London, London, UK; 12 Faculty of Physics and Astronomy, UHD, Heidelberg, Germany; 13 Hertie Institute for AI in Brain Health, University of Tübingen, Tübingen, Germany

**Keywords:** Psychology, Mathematics and computing

## Abstract

Ecological momentary assessment (EMA) enables fine-grained tracking of affective and behavioral states in daily life. Accurately forecasting these states and their responses to interventions can guide adaptive mental health strategies. Network-based models are commonly used to capture such psychological dynamics, but most existing approaches make linear assumptions, and are rarely evaluated on forecasting performance. More flexible nonlinear models could better match evidence that psychological processes unfold in nonlinear, context-dependent ways and may offer superior predictive accuracy, but their internal dynamics are typically less interpretable. Here, we benchmarked a spectrum of models across three 40 day micro-randomized trials (*N* = 145), spanning linear network models, nonlinear state-space models (SSMs) based on piecewise-linear recurrent neural networks (PLRNNs), and Transformers. Three key findings emerged. First, PLRNNs provided the most accurate forecasts of spontaneous and intervention-driven EMA dynamics. Second, their latent-network structure yielded psychologically interpretable connectivity patterns, identifying affective nodes such as *relaxed* as high-impact influence points. Third, the inferred dynamics allowed simulating future perturbations, establishing a direct link between psychological network structure, forecasting, and intervention planning. Model performance remained robust under real-time retraining and incomplete data, indicating that nonlinear SSMs offer a practical and interpretable foundation for real-time control in digital mental health.

## Introduction

Digital mental health interventions, mobile mental health (mHealth) applications in particular, provide a cost-effective and accessible way for individuals with mental health conditions to access evidence-based mental health services^1,2^, bridge waiting periods before psychotherapy^3,4^, or supplement ongoing treatment in blended care models^5^. These apps increasingly incorporate Ecological Momentary Assessments (EMA) to capture real-time psychological states and behavior, enabling the continuous tracking of mental health in daily life to inform the delivery of intervention components that are more tailored to moment, context, and person, including Ecological Momentary Interventions (EMIs) or Just-in-Time Adaptive Interventions (JITAIs)^6–10^. EMA data - such as self-reported mood, anxiety, stress, worry, or arousal levels - are essential for identifying critical moments when interventions may be most effective, such as detecting early signs of depression or escalating anxiety that warrant timely coping strategies^7,11,12^.

A growing body of research suggests that EMA variables are not isolated indicators but interact within complex psychological networks^13,14^. This network approach aligns closely with the perspective that psychological functioning is best understood as a dynamical system (DS)^15,16^, where individual states influence each other across multiple time scales^17–20^. For example, network models have been employed to elucidate how therapy attenuates the mutual interactions between anxiety and avoidance^21^, how transdiagnostic irritability is driven by frustration^22^, how more generally emotional states can shift nonlinearly between steady and oscillatory patterns^23^, and how the interactions between internal and external factors can give rise to psychiatric symptoms^24^.

Moreover, they offer insights into how EMI can promote adaptive change over time^25^. These insights are increasingly being used to tailor mHealth interventions to individual needs, enhancing personalization and clinical relevance^11,25–28^.

Most recent research uses linear methods such as vector autoregressive models^29^, hierarchical linear modeling^30,31^, and mixed-effects models^32^, to analyze EMA data. These methods are widely used because they offer interpretability and simplicity in modeling temporal relationships between psychological variables.

However, psychological time series often show clear signatures of nonlinear dynamics, including multistability^33^ and phase transitions linked to sudden mood shifts, depressive episode onset, and recovery trajectories^34^. Linear models on the other hand assume fixed, proportional relationships between variables^35^ and are fundamentally limited in their ability to capture such dynamics. This raises concerns about their suitability for forecasting psychological states, especially in contexts where accurate, real-time prediction is essential for adaptive intervention, such as in model-predictive control frameworks.

Here, using three independent and comparable datasets, we robustly demonstrate that forecasting models accounting for nonlinear interactions between dynamic variables significantly outperform linear approaches in predicting psychological states, providing a more valid and realistic generative framework. In addition to improved predictive accuracy, these nonlinear models yield interpretable network structures, shedding light on the underlying dynamics of psychological state interactions. Specifically, we show that certain classes of nonlinear dynamical models not only illuminate the architecture of psychological networks but could also help to identify the most promising targets for intervention. Taken together, these findings highlight the critical importance of nonlinear modeling for guiding the personalized delivery of EMIs. The modeling results are highly replicable and generalize to real-time deployment.

## Results

### Forecasting psychological states from EMA and EMI data

**Fig. 1:**
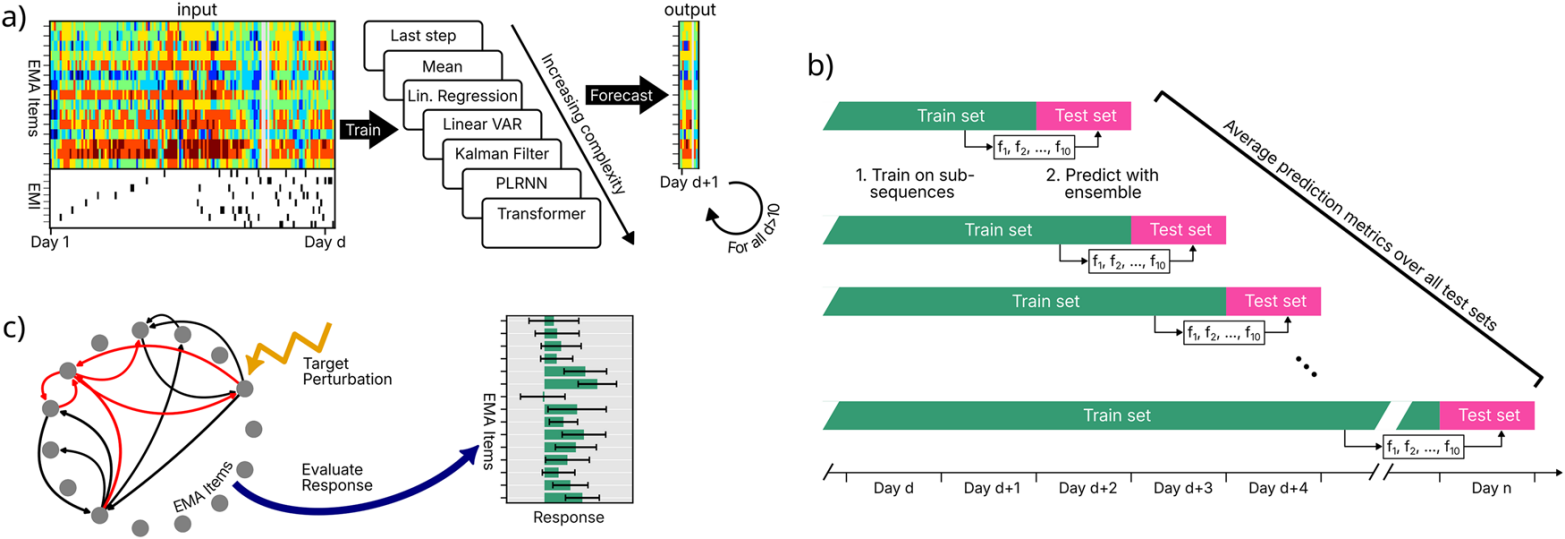
Study procedure. **a** Modeling procedure. Individual EMA and EMI time series up to day *d* (left) served as input to a range of forecasting models with increasing complexity (middle), which were then used to forecast EMA trajectories for day *d* + 1 (right). **b** Online model training/evaluation procedure. For each day *d*, the train set included the entire time series up to day *d*, while the test set consisted of data from day *d* + 1. For each train set, 10 models were trained with independent random initializations, and their average prediction was used as the forecast. Evaluation metrics were computed across all test days. **c** Targeted perturbation procedure. After fitting the computational network models, we simulated node-level perturbations targeted at specific EMA network items. Predicted effects of these inputs were then quantified at the item level using cumulative impulse responses. Figure created with Inkscape.

While EMAs enable real-time assessment and monitoring of psychological states and timely interventions in mHealth applications including EMI and JITAI, the predominant use of linear models has constrained their ability to capture the complex, nonlinear interactions inherent to psychological processes. To overcome these limitations, we systematically evaluated the predictive accuracy and interpretability of various forecasting models using three unique EMA + EMI datasets collected over 40 days, with a total sample size of *N* = 145, in the AI4U living lab^36^. More details on the project can be found in refs. ^37,38^. More specifically, we employed an online training/evaluation scheme which imitates how such models would be deployed in a real-world scenario (see Fig. 1a, b). We compared a set of model classes ranging from simple, static baselines to advanced dynamic network models, including first order vector autoregressive models (VAR(1)), linear state-space models (Kalman filters), nonlinear state-space models (SSMs) incorporating latent piecewise-linear recurrent neural networks (PLRNN)^39–41^, and autoregressive Transformer architectures^42,43^ (see Supplementary Fig. 1 and Methods section 4, equations (3)-(9) for rationale behind these choices). Hyperparameter tuning was conducted exclusively on the first dataset (sample 1), while sample 2 and sample 3 served as independent test sets.

### Nonlinear models outperform linear approaches

**Fig. 2:**
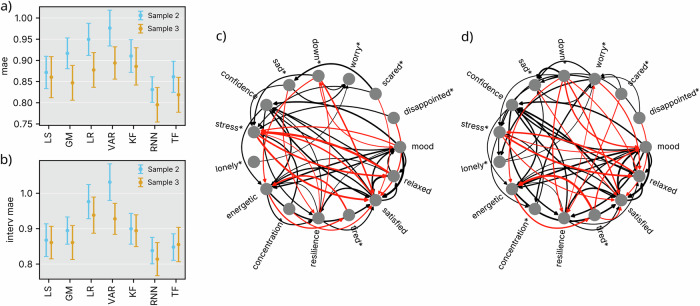
Forecasting and network results. **a** Mean Absolute Error (MAE) of predicted absolute EMA values across all models (LS=last step, GM=global mean, LR=linear regression, VAR=vector autoregressive model of order 1, KF=Kalman filter, RNN=PLRNN, TF=Transformer). Mean and standard error are displayed. **b** MAE of predicted absolute EMA values immediately succeeding an EMI, reflecting the specific ability of predicting intervention-induced shifts. **c**, **d** PLRNN models depicted as ideographic networks. Arrow width depicts mean edge strength over all participants. Positive connections are shown in black, negative connections in red. Only those are shown that were significantly different from 0 (Bonferroni corrected for multiple comparisons at *p*_Bonf_ < 0. 01). For visualization purposes, negatively poled items were not recoded and marked with an asterisk. **c** Networks from sample 2. **d** Networks from sample 3. Figure created with Matplotlib^63^ and Inkscape.

Across the two independent replication samples (sample 2 with 48 and sample 3 with 51 participants, respectively), nonlinear forecasting models consistently outperformed linear methods. Specifically, the PLRNN model significantly improved prediction accuracy of daily psychological state fluctuations, achieving lower mean absolute errors (MAE; sample 2 = 0.831, sample 3 = 0.795, Fig. 2a) compared to VAR models in both samples, and compared to Kalman filter models in sample 2. Detailed results can be found in Table 1. Notably, linear models even performed significantly worse than simple baseline approaches, such as using the previous time step or the overall expectation as predictors. Adding to that, PLRNN models also outperformed linear models in predicting EMA states immediately following an EMI (sample 2 = 0.838, sample 3 = 0.814, Fig. 2b), highlighting its potential for informing interventions. For all dynamical models, MAE increased with forecast length (c.f. Supplementary Fig. 2).

**Table 1:** Evaluation metrics for all tested models in samples 2 and 3

Sample	Model	MAE	Interv. MAE	MAE compared to PLRNN
2	Last step	0.872 ± 0.0382	0.868 ± 0.0461	*t*(47) = 2.14, *p*_Bonf._ = 0. 228
	Global Mean	0.917 ± 0.0363	0.895 ± 0.0384	*t*(47) = 5.71, *p*_Bonf._ < 0. 001
	Linear Regression	0.950 ± 0.0379	0.976 ± 0.0482	*t*(47) = 8.44, *p*_Bonf._ < 0. 001
	VAR(1)	0.976 ± 0.0422	1.031 ± 0.0515	*t*(47) = 4.81, *p*_Bonf._ < 0. 001
	Kalman filter	0.910 ± 0.0385	0.900 ± 0.0434	*t*(45) = 3.86, *p*_Bonf._ = 0. 002
	PLRNN	**0.831** ± **0.0300**	**0.838** ± **0.0374**	−
	Transformer	0.861 ± 0.0367	0.848 ± 0.0374	*t*(47) = 1.88, *p*_Bonf._ = 0. 401
3	Last step	0.860 ± 0.0485	0.861 ± 0.0459	*t*(50) = 2.59, *p*_Bonf._ = 0. 075
	Global Mean	0.847 ± 0.0410	0.861 ± 0.0481	*t*(50) = 6.56, *p*_Bonf._ < 0. 001
	Linear Regression	0.877 ± 0.0410	0.938 ± 0.0511	*t*(50) = 8.31, *p*_Bonf._ < 0. 001
	VAR(1)	0.894 ± 0.0381	0.928 ± 0.0438	*t*(50) = 5.59, *p*_Bonf._ < 0. 001
	Kalman filter	0.886 ± 0.0439	0.894 ± 0.0449	*t*(45) = 2.26, *p*_Bonf._ = 0. 172
	PLRNN	**0.795** ± **0.0407**	**0.814** ± **0.0469**	−
	Transformer	0.819 ± 0.0411	0.855 ± 0.0485	*t*(50) = 2.75, *p*_Bonf._ = .050

Mean and standard error are displayed. Additional paired *t*-test results for the comparison of the absolute score MAE between each model and the best performing model (PLRNN), with Bonferroni-corrected *p-*values. Lower degrees of freedom in the Kalman filter are a result of non-convergence for a few subjects.

A dedicated stability analysis, in which we estimated the maximal Lyapunov exponent using a QR-based method^44,45^, showed that PLRNN dynamics were predominantly stable and that accurate forecasts were generated mainly by transient trajectories evolving toward a limiting set rather than by unstable or chaotic behavior (Supplementary Fig. 3).

### Enhanced interpretability of nonlinear network models

Beyond predictive performance, the PLRNN yielded a larger number of statistically significant edges in the inferred networks compared to the other models, yielding insight into interpretable dynamical mechanisms. Obtaining ideographic networks from this nonlinear model was facilitated by its specific architecture. Because the PLRNN model incorporates a piecewise-linear activation structure, local derivatives can be analytically derived (see Methods 4). This capability enabled us to transparently reconstruct interpretable psychological network dynamics directly from the model. Figure 2c, d depicts the effective connectivity of the PLRNNs in observation space, delivering directly interpretable behavioral contingencies, consistent across the sample, Bonferroni corrected for multiple comparisons.

Across all participants, several EMA items exhibit significant connectivity - for example, *stress* and *relaxed* are mutually inhibitory, so are *tired* and *energetic*. In contrast, *mood* and *relaxed*, as well as *confidence* and *satisfied* are mutually excitatory. While significance levels differed between samples, the overall connectivity pattern remained largely consistent across samples and was intuitively interpretable, exhibiting high face validity. In contrast, connectivity inferred from VAR(1) and Kalman filter models was notably more sparse (Supplementary Fig. 4), with the VAR(1) model exhibiting almost no significant edges. Moreover, although Transformer architectures achieved reasonable forecasting performance, they cannot be interpreted as ideographic networks - analogous to many other deep neural networks - and require longer training times, making them less suitable for real-time deployment in e.g. digital mental health interventions (see Supplementary Fig. 5).

### Identifying targets for personalized intervention

**Fig. 3:**
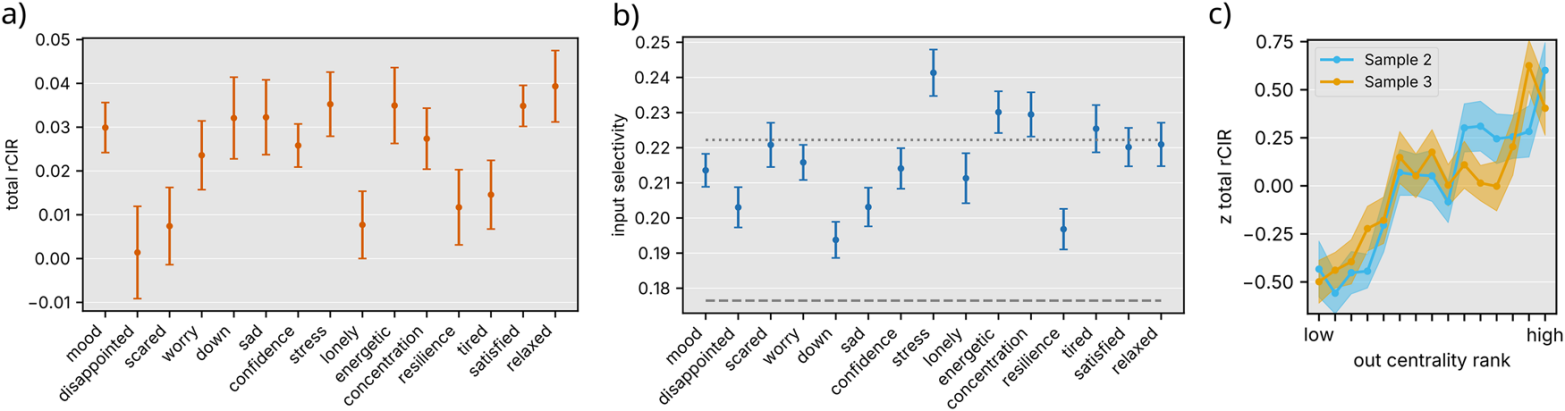
Individual node perturbation response. **a** Total rCIR to inputs targeting each individual EMA item. Mean and standard error are displayed. **b** Input selectivity score for each node, quantifying how strongly a given item is targeted relative to the others. If all items were perturbed equally (no selectivity), the score would be 1/*n* = 0.067. The dashed line indicates the expected score when one item is perturbed three times more strongly than the others, and the dotted line when it is perturbed four times more strongly. **c** Total rCIR (standardized within subjects) to inputs targeting specific nodes, plotted against the out-degree centrality rank of these nodes (higher rank number = higher centrality). The solid line indicates mean rCIR, shaded areas the standard error of the mean. Figure created with Matplotlib^63^ and Inkscape.

Exploiting the PLRNN’s explicit latent-network formulation, we next examined how targeted disturbances of single psychological states ripple through the network to shape overall mental-health trajectories (Fig. 3a, b). Our analysis of the network dynamics revealed that perturbing certain EMA states exerts a disproportionately strong influence. In particular, nodes such as *relaxed*, *satisfied*, *energetic* and *stress* exhibited high relative cumulative impulse responses (rCIRs), marking them as promising targets for intervention. In contrast, other states—such as *disappointed*—showed weaker ripple effects, suggesting they may be less effective.

The computed rCIR metric (see Methods 4, Eqn. (1)) also carried an intuitively interpretable meaning in terms of the network’s structure: states with high rCIRs tended to occupy more central positions in the psychological network, exerting influence over multiple interconnected nodes (Spearman’s *ρ* = 0.89/0.83 in sample 2/3, *p* < 0. 001, see Fig. 3c). When directed to the nodes with lowest out centrality, external inputs induced a significantly smaller absolute rCIR than when directed to the nodes with highest weighted out-degree centrality (*t*(55) = − 4.51, *p* < 0. 001 in sample 2; *t*(58) = − 4.87, *p* < 0. 001 in sample 3), indicating the importance of strategically selecting intervention targets based on network centrality and rCIRs. However, rCIR has the advantage of quantifying predicted effects on a single-item resolution, while centrality only provides a system-wide measure. Crucially, the ability to specifically target isolated EMA nodes for mechanistic insight stems from the PLRNN’s unique architecture, enabling analytically tractable perturbation analyses. These findings provide an evidence-based framework for tailoring interventions to individual psychological network structures, that may optimize the effectiveness of EMIs, and help identifying candidate intervention targets.

**Fig. 4:**
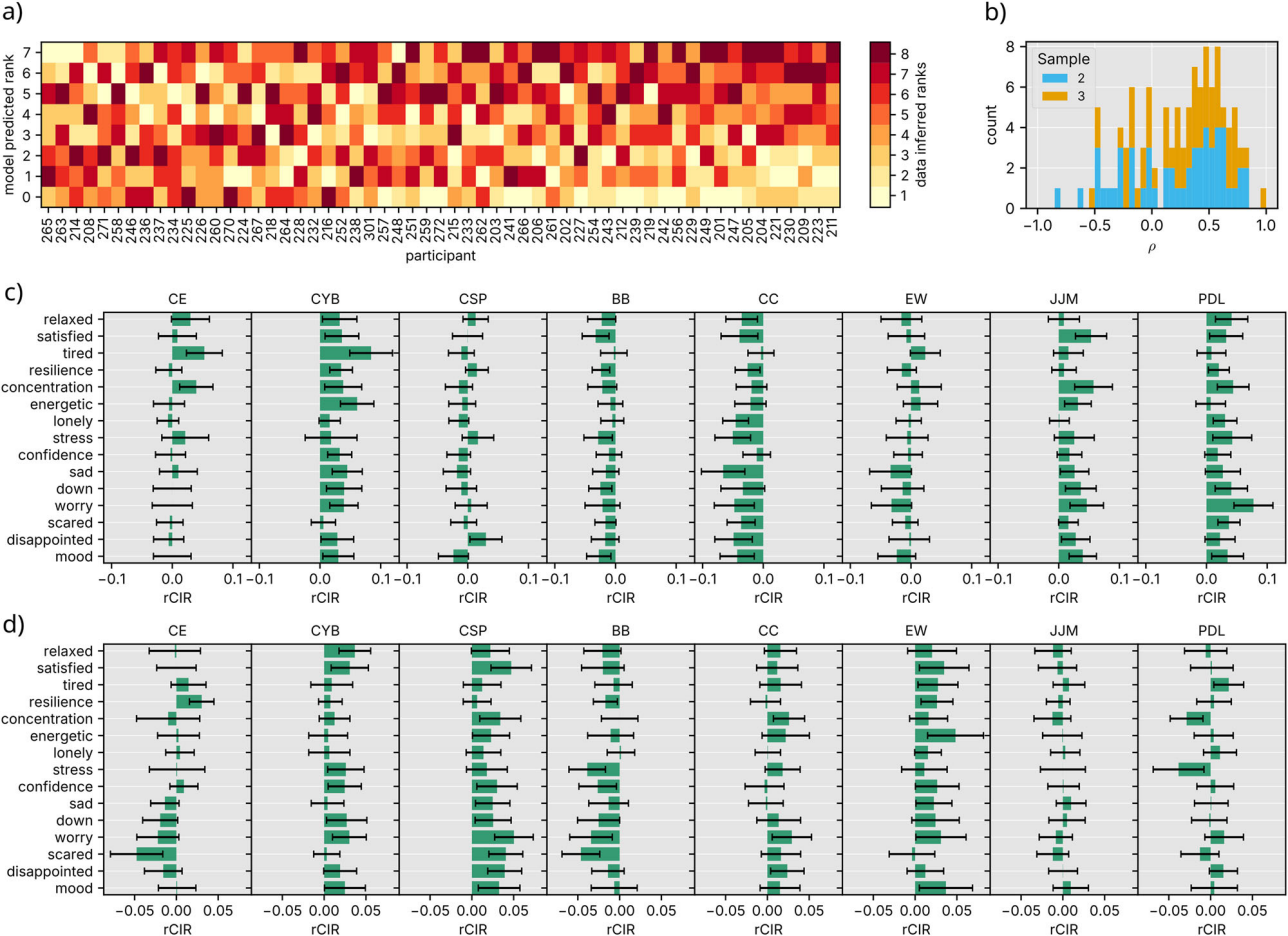
EMI perturbation response. **a** Empirical EMI ranks sorted by predicted EMI ranks for each participant of sample 3, ordered by weighted Spearman correlation between predicted and empirical ranks. For participants on the right side, correlations are highest (rCIRs most accurately predict proximal EMI effects). Predicted effectiveness was measured by rCIR_1_, while empirical effectiveness was assessed as the one-step difference in EMA trajectories after the occurrence of an EMI. A perfect prediction would result in the order of colors shown in the right bar. Two participants were excluded because they did not receive all EMI types. **b** Distribution of weighted Spearman correlations between predicted and empirical EMI ranks for both samples. **c** rCIR_7_ for each EMI in sample 2. Mean and standard error are displayed. EMI types: CE=Compass of emotions, CYB=Counting your breath, CSP=My calm and safe place, BB=Breathing with breaks, CC=My compassionate companion, EW=Emotion as a wave, JJM=Journal of joyful moments, PDL=Positive data log. **d** Same for sample 3. Figure created with Matplotlib^63^ and Inkscape.

We also applied this novel approach to forecast and evaluate the effects of the administered EMIs by perturbing the inferred networks and computing their rCIR trajectories over a 24 h period (c.f. Fig. 1c). Although direct validation is challenging due to the influence of multiple uncontrolled external factors in the empirical EMA time series, the models again produced plausible predictions: most EMIs were associated with either significantly positive, selectively positive, or neutral effects on mental health (see Fig. 4c, d). Only one EMI was linked to adverse predicted outcomes per sample.

While we cannot easily validate these forecasts, we evaluated how reliably individual-level predictions of rCIR effects matched empirical proximal (one-step) effects (Fig. 4b). To account for varying numbers of EMIs per perticipant, we used weighted Spearman correlations between predicted and empirical effects. Approximately 14.2% of participants showed small (0.1 < *ρ*_*w*_ < 0.3), 23% moderate (0.3 < *ρ*_*w*_ < 0.5), and 31% large (*ρ*_*w*_ > 0.5) effects. This indicates the ability to personalize predictions across all eight interventions by identifying both the most effective EMI and its relative ranking for each individual (Fig. 4a; Supplementary Fig. 6).

Group-level correlation estimates were then obtained by Fisher-*z* transforming individual correlations prior to averaging and computing standard errors. Significance was assessed via a hierarchical bootstrap that resampled subjects and, within subjects, EMI-type effect pairs with replacement, yielding a bootstrap distribution of mean correlations from which confidence intervals and *p*-values were assessed. Across participants, the mean weighted correlation was significantly greater than zero (${\bar{\rho }}_{w}=.253/.327,p=.03/.002$ in sample 2/3), and results remained stable when excluding individuals with few EMIs, indicating that correlations were not driven by sparsely sampled participants (Supplementary Fig. 7). Moreover, compared to a null distribution of near-zero correlations generated by a participant-wise null-timing bootstrap, in which EMI timestamps were repeatedly shuffled while preserving EMA time-courses, the observed real-timing correlations were significantly higher (empirical *p* < 0. 001 in both samples, Supplementary Fig. 8), indicating that predictive performance reflects intervention-specific signal rather than generic temporal structure.

### Forecasting implications for real-world study designs

Several findings also carry important implications for real-world study design. Nonlinear models such as the PLRNN retained high predictive accuracy even in the presence of missing data, underscoring their practical applicability (Supplementary Fig. 9b). Notably, data completeness—defined as a high proportion of non-missing responses—proved more critical for PLRNN performance than the overall length of the time series (Supplementary Fig. 9a, b). This relationship varied by model type; for example, the VAR(1) showed a stronger dependence on data length.

Forecasting performance also systematically varied by EMA item type, with psychological variables generally exhibiting higher predictive accuracy than physical ones. For instance, the MAE for *tired*—which arguably reflects a more physical or somatic state - was approximately twice that of *resilience* (Supplementary Fig. 10c). The rank ordering of prediction errors was nearly identical for the PLRNN and Kalman filter models, with only two items differing in rank (see Supplementary Fig. 10a–c). Moreover, these patterns were once more highly stable across samples, with Spearman rank correlations of *ρ* = 0.925 (*p* < 0.001).

Consistent with expectations, non-stationary items negatively impacted forecasting (*t*(1483) = − 2.62, *p* = 0.004 across both samples), highlighting the importance of adaptive methods. Nonetheless, non-stationarity was limited over the 40 day window: in samples 1-3, 82%, 77%, and 80% of participants, respectively, had ≥ 14 (out of 15) items classified as stationary (augmented Dickey-Fuller test at *α* = 0.05).

Finally, incorporating additional activity-related external inputs improved forecasting performance compared to using only EMI as inputs—an effect observed specifically for the PLRNN (*t*(36) = 4.02, *p* < 0. 001 across both samples), but not significantly for other network models (see Supplementary Fig. 11). These findings suggest that combining a flexible, nonlinear forecasting model with relevant contextual information can further enhance predictive accuracy.

## Discussion

This study demonstrates, for the first time to the best of our knowledge, that nonlinear dynamical models markedly outperform conventional linear network approaches in predicting momentary psychological states from multivariate EMA data, and that their ability to incorporate EMI as external inputs may establish a robust methodological platform for next generation adaptive digital mental health interventions. By leveraging a series of intensive longitudinal datasets collected under ecologically valid conditions, we demonstrate that nonlinear SSMs—particularly the PLRNN—outperform traditional linear approaches in predictive accuracy and interpretability.

A key insight emerging from our analysis is that psychological processes, when modeled as interacting elements within a dynamic network, yield both more accurate forecasts and more meaningful representations of underlying mental states. This finding supports a growing consensus that mental health cannot be adequately captured through static or linear models, and instead reflects complex, nonlinear interactions evolving over time^16,46–48^. Importantly, these interactions are not only quantifiable but also exploitable: by embedding psychological states in a generative latent space, the PLRNN enables precise perturbation analyses that can identify high-impact intervention targets, laying the basis for model-based predictive control^25,49^.

Most existing EMA studies rely on linear models such as VAR(1) or hierarchical regressions, which, while accessible and interpretable, cannot capture nonlinear transitions or latent influences. In contrast, the PLRNN not only accommodates such mechanisms but does so with moderate effective complexity. Although its training procedure is more elaborate than that of linear models, the number of free parameters is partly comparable to, and in some configurations even smaller than, that of VAR(1) and Kalman filter models, and per-subject training times remain on the order of only a few seconds (Supplementary Fig. 5). Moreover, PLRNN ensembles retain strong predictive performance even when trained on substantially shorter and sparser time series—central to real-world sparse data settings^50,51^—whereas linear models degrade more rapidly as data become scarce (Supplementary Fig. 9), suggesting that the additional flexibility of the PLRNN translates into greater data efficiency and robustness to missingness.

In practice, we can envision a hybrid deployment strategy: inference and feedback generation can run locally on a mobile device using pre-trained parameters, whereas full model retraining would typically occur server-side or during idle device periods. Preliminary comparisons between server- and smartphone-class processors even suggest that limited on-device retraining is technically feasible for models of this size, but comprehensive profiling of energy, memory, and latency requirements remains an important direction for future work. Taken together, these results indicate that, in our setting, the performance and robustness gains of the PLRNN clearly outweighed its additional computational cost, making the trade-off between model complexity and predictive benefit advantageous overall. We acknowledge, however, that applications at much larger scale or under strict real-time constraints may require additional optimization.

Beyond prediction, the nonlinear models revealed interpretable psychological networks whose structure remained consistent across participants and datasets. These networks exhibited face-valid patterns – for instance, stress and relaxation showed mutual inhibition—while linear models such as VAR produced almost no significant links. This lack of significant VAR(1) links likely reflects a combination of model and data limitations. Classical VAR models operate purely in observation space and cannot represent latent moderating states or nonlinear interactions, so any higher-order or latent-driven dynamics are forced into a single linear coefficient between observed items. In our relatively short, noisy EMA time series, this may lead to weak and unstable estimates that rarely reach significance after correction. By contrast, the Kalman filter and the PLRNN can absorb such dynamics into their latent state, and the PLRNN additionally captures nonlinear interactions, resulting in stronger and more consistently detectable effective connections at the observation level. Taken together, our incremental model comparison suggests that introducing a latent space is particularly beneficial for prediction accuracy. Adding nonlinear dynamics, in turn, yielded a denser pattern of statistically significant edges at the observation level, which may reflect an improved ability to capture structured dependencies between items.

The PLRNN’s capacity to map such networks stems from its piecewise-linear structure, which allows for the reconstruction of locally linear approximations of psychological dynamics that are both mathematically tractable and behaviorally interpretable, presenting a methodological advancement in psychological network anaylsis^52^.

Crucially, these network representations served to inform potential interventions. By systematically perturbing individual network nodes, we identified states such as *relaxed* and *satisfied* as high-leverage points whose targeted modulation—e.g., through EMI components—elicited widespread effects across the system. The strength of these effects scaled with network centrality. Indeed, forecasts of real EMI effects across a 24 h window aligned with these insights, showing that most EMIs had either positive or neutral predicted outcomes, consistent across independent datasets.

We also identified key determinants of prediction quality. Forecasting accuracy varied by item type, with psychological variables (e.g., mood, anxiety) showing higher predictability than physical ones (e.g., tiredness), likely due to stronger embedding within the internal psychological network. Moreover, data completeness, rather than time series length, proved more important for prediction—particularly in nonlinear models—highlighting a clear design recommendation for future studies: prioritize sampling consistency over duration.

Finally, our results also highlight the importance of interpreting improvements over simple baselines with care. The very simple “Last Step” persistence model performed surprisingly well for short-term forecasts, in some cases approaching the accuracy of much more flexible architectures such as the Transformer. This is consistent with the strong autocorrelation structure of EMA time series. When successive assessments are highly correlated, the most recent observation already carries substantial information about the immediate future, making persistence a stringent benchmark that should be routinely reported^53^. Conceptually, however, such baselines are fundamentally limited: they cannot incorporate exogenous inputs, simulate counterfactual trajectories, or support intervention planning. In contrast, generative models provide explicit latent dynamics that can be perturbed, rolled out over longer horizons, and conditioned on hypothetical inputs. In extended analyses with longer prediction windows, these advantages become increasingly apparent (see e.g., Supplementary Fig. 12).

Taken together, the findings of improved predictive accuracy and interpretable insights into predictive mechanisms pave the way for personalized feedback and intervention designs based on nonlinear SSMs. However, while this work represents a significant advance, future studies should extend these models to incorporate multimodal inputs (e.g., passive sensing, geospatial data, clinical metadata, or biophysiological data)^54–56^, explore adaptive model updating in long-term deployments^50^, and refine the integration of control theoretic principles for intervention optimization (e.g.,^25^), perhaps employing reinforcement learning techniques^57^. Nevertheless, despite the constraints of limited data, our findings demonstrate that nonlinear SSMs offer a powerful approach for accurately forecasting psychological states and intervention effects, laying the groundwork for real-time model-predictive control of mental-health interventions.

## Methods

### Data and study design

We analyzed three independent sequentially conducted micro-randomized trials (AI4U samples 1-3; *n* ≈ 60 per sample) from which we included *n* = {46, 48, 51} participants, totaling *N* = 145 (see Supplementary Table 1)^36^. Each study spanned 40 days and comprised a 10-day training phase followed by a 30 day assessment phase.

In each study, participants received EMA surveys and EMI exercises (from a set of eight options) multiple times per day. After screening, they completed a 10 day training phase in which each EMI was presented twice, alongside eight EMA surveys per day at random times within a user-defined interval. During the remaining 30 days, six EMA surveys were delivered per day. After each one, participants could request an EMI ("interactive tasks”), and one additional EMI was delivered daily at a user-defined time (“consolidation task”). At each EMI delivery, an exercise was selected from the eight interventions either randomly or via a selection algorithm (50% probability each), with the option of a null intervention (see also^36^).

The EMA protocol analyzed comprised 15 well-being items rated on 1-7 Likert scales (reverse-scored as required; Supplementary Table 2) covering affective, cognitive and motivational states, plus six auxiliary covariates (Supplementary Table 3, see covariates sub-analysis in Supplementary Fig. 11). This design yielded up to 260 multivariate EMA observations per participant over 40 days, and, depending on interactive acceptances, on the order of ~ 200 EMIs (realized counts are reported in Supplementary Table 1). The set of EMI used in this study focused on resilience, emotion regulation, and stress reduction^58^.

For each participant *s*, EMA responses were arranged in a matrix ${{\bf{X}}}_{s}\in {{\mathbb{R}}}^{T\times n},$ where *T* is the number of recorded time points and *n* the number of EMA items. The *i*^th^ column represents the time course of item *i*, and the *t*^th^ row contains all EMA responses at time point *t*. For brevity, the subscript *s* is omitted in the following. EMI deliveries were encoded in a binary matrix **U** ∈ {0, 1}^*T*×*m*^, where *m* (here 8) is the number of admissible EMI. An entry of 1 in row *t* and column *i* indicates that intervention *i* was delivered at time *t*. Because interactive tasks were delivered immediately after an EMA, rows of **U** were aligned with those of **X**; consolidation tasks, delivered independently of EMA, were assigned to the most recent preceding EMA time point to maintain temporal alignment.

Prior to modeling, for training data only, EMA items were smoothed using a causal right-truncated Gaussian kernel (4 h window, *σ* = 1.5 h) to accommodate irregular sampling^59,60^. After smoothing, each EMA item was mean-centered using training-set statistics. Test data remained unsmoothed and centering used training-set statistics; ensuring strict separation between training and test set. All analyses followed a unified pipeline linking data preprocessing, model training, forecasting, and evaluation (see also Fig. 1).

### Forecasting and model evaluation protocol

We adopted an evaluation framework mirroring a real-time pipeline, in which forecasting models are retrained each night on all data up to the first EMA of the day and used to predict the subsequent seven observations - amounting to 1 day - at the time of EMI selection^36^. As depicted in Fig. 1b, this “rolling-origin” design ensured strict temporal causality and avoided information leakage by keeping all training data strictly prior to the evaluation window. Eligible test days were those occurring on or after day 11 with at least three completed EMAs including the first of the day; days failing these criteria were excluded to guarantee stable estimation of daily error metrics. Participants without any eligible test day were not included in the forecasting analyses. For each eligible day, data prior to the first EMA defined the training set, and that day’s remaining observations constituted the test set. Predictive accuracy was assessed by prediction error on absolute scores (MAE). MAE was computed separately for (i) all EMA points and (ii) EMAs immediately following an EMI to assess model sensitivity to intervention-related changes. For each participant, MAE was obtained by first averaging absolute errors across EMA items at each non-missing time point, then across time points within each eligible test day, and finally across all test days; group-level comparisons were based on these subject-level MAE values. The intervention-specific MAE was defined analogously, but restricted to EMA observations immediately following an EMI.

Models whose fitting procedures involved non-deterministic optimization were trained ten times with random initializations, and forecasts were based on the ensemble mean. Sensitivity analyses using ensembles of 10-20 models yielded nearly identical mean predictive performance at very similar variance (see Supplementary Fig. 13), indicating that an ensemble size of 10 provides stable estimates while keeping computational cost moderate. All model fitting and evaluation procedures were executed using identical computational settings (PyTorch 2.1, AMD Epyc 7452 CPU).

### Modeling

To benchmark forecasting performance, we implemented a continuum of models that spanned simple baselines and interpretable linear dynamics to high-capacity nonlinear architectures (see Supplementary Fig. 1 and extended Methods 4). All models shared the same multivariate input-output structure (Fig. 1a), ensuring that differences in performance reflected model dynamics rather than preprocessing or feature engineering. Hyperparameters for each model were optimized by grid search on sample 1 to minimize MAE (Supplementary Table 4); the resulting configurations were then fixed and applied without further tuning to samples 2 and 3 for all comparative analyses.

We roughly distinguish between three model classes. *Non-dynamic baselines* included (i) a last-observation carry-forward ("Last Step”) model, (ii) a participant-specific global mean predictor, and (iii) a linear regression model with external (i.e., EMI) inputs. *Linear dynamical models* captured temporal dependencies either directly in observation space via a first-order vector autoregression (VAR(1)) with ridge-regularized coefficients, or in latent space using a linear state-space formulation (Kalman filter) with learned transition and observation matrices. *Nonlinear dynamical models* extended these approaches to capture complex, state-dependent trajectories: a PLRNN trained via generalized teacher forcing^40^, and an autoregressive Transformer with causal self-attention and position-encoded temporal context.

Successive increases in model complexity were often achieved by altering a single model component at a time—such as adding external inputs, auto-regression, latent states, or introducing nonlinearity—thereby allowing systematic assessment of how each ingredient contributed to forecasting accuracy (see Supplementary Fig. 1, and the “Extended methodological details” section).

### Network connectivity

To quantify how psychological states influence one another across time, we derived local observation-space connectivity by computing the Jacobian of the conditional expectation ${\mathbb{E}}[{{\bf{x}}}_{t}| {{\bf{x}}}_{t-1}={\bf{x}}]$ with respect to **x**. In the VAR(1) model, the Jacobian reduces to the coefficient matrix **A**, so each element *A*_*ji*_ directly quantifies the linear influence of variable *i* on variable *j* across time^14^. For the Kalman filter, the effective observation-space connectivity is $\frac{\partial {\mathbb{E}}[{{\bf{x}}}_{t}| {{\bf{x}}}_{t-1}={\bf{x}}]}{\partial {\bf{x}}}={\bf{B}}{\bf{A}}{{\bf{G}}}_{KF},$ where ${{\bf{G}}}_{KF}={\Sigma }_{zx}{\Sigma }_{xx}^{-1}$ is the posterior sensitivity of **z**_*t*−1_ to **x**_*t*−1_, derived from the joint Gaussian over (**z**_*t*−1_, **x**_*t*−1_) under stationarity. The latent covariance Σ_*zz*_ is obtained from the discrete Lyapunov equation, and Σ_*zx*_ and Σ_*xx*_ follow as Σ_*zx*_ = Σ_*zz*_**B**^⊤^ and Σ_*xx*_ = **B**Σ_*zz*_**B**^⊤^ + Γ. For the PLRNN, ReLU activations partition the latent space into linear regions $(\{{\Omega }_{j}\})$, each with a local Jacobian ${{\bf{J}}}^{({\Omega }_{j})}=\frac{\partial {{\bf{z}}}_{t}}{\partial {{\bf{z}}}_{t-1}}$. Hence, $\frac{\partial {\mathbb{E}}[{{\bf{x}}}_{t}| {{\bf{x}}}_{t-1}={\bf{x}}]}{\partial {\bf{x}}}\approx {\bf{B}}\,{{\bf{J}}}^{({\Omega }_{j})}{{\bf{G}}}_{{RNN}},$ where Ω_*j*_ is determined by the latent state inferred from **x**_*t*−1_. We estimate the PLRNN recognition gain **G**_*RNN*_ by generalized least squares, ${{\bf{G}}}_{RNN}^{{\rm{(GLS)}}}={({{\bf{B}}}^{\top }{{\mathbf{\Gamma }}}^{-1}{\bf{B}})}^{-1}{{\bf{B}}}^{\top }{{\mathbf{\Gamma }}}^{-1},$ with **Γ** the observation covariance, implicitly corresponding to a flat prior on **z**_*t*−1_.

Connectivity matrices for all models were evaluated along each participant’s EMA trajectory and time-averaged (using the model ensemble trained on the longest available training set) to obtain participant-level networks.

### Network perturbations

To assess the impact of targeted interventions, we quantified how external inputs **u** altered model predictions using the seven-step CIR. For a given initial observation **x**_0_, we projected into latent space using the recognition mapping **G**_*RNN*_**x**_0_ and iterated the latent dynamics *f* under input **u**. The seven-step CIR (corresponding to roughly a 24-h horizon) was defined as

1 $${\mathrm{CIR}}_{7}({\bf{u}},{{\bf{x}}}_{0})=\mathop{\sum }\limits_{t=1}^{7}\left({\bf{B}}\,{f}^{t}({{\bf{G}}}_{RNN}{{\bf{x}}}_{0},{\bf{u}})-{{\bf{x}}}_{0}\right),$$

where ${f}^t$ denotes *t* successive applications of the latent transition function, and **B** the mapping into observation space. To isolate the input-induced component from the system’s spontaneous evolution, we computed the relative CIR (rCIR) by subtracting the no-input trajectory, CIR_7_(**0**, **x**_0_)^61^. CIRs and rCIRs were averaged across representative initial conditions, including the null vector and all standard basis vectors.

Inputs either corresponded to the actual EMIs used in the study or to synthetic, node-specific perturbations. For the latter, we constructed input vectors whose immediate effect at the observation level, **Du** with **D** = **BC** (**C** mapping inputs to states, see extended Methods 4), was maximally aligned with a single target EMA item (through maximizing cosine similarity), yielding the most selective direction permitted by the model (c.f. Supplementary Fig. 14). Because **D** generally mixes items, perfect specificity is not achievable; we therefore quantified selectivity using

2 $${s}_{j}=\frac{| {({\bf{D}}{\bf{u}})}_{j}| }{\mathop{\sum }\nolimits_{i = 1}^{n}| {({\bf{D}}{\bf{u}})}_{i}| },$$

which equals 1 for a perfectly item-specific perturbation and approaches 1/*n* when effects are uniformly distributed across items (see Fig. 3b).

Finally, we summarized a node’s overall influence by its total rCIR (the sum of rCIR effects across all EMA variables) and related this measure to its weighted out-degree centrality—defined as the total absolute weight of its outgoing edges—to identify high-impact candidates for personalized interventions.

### Determinants of forecasting performance

We first assessed item-level predictability by correlating each EMA item’s MAE and directional accuracy with its mean and stationarity using Spearman’s rank correlation. To characterize performance under limited data, we identified participants with at least ten eligible test days after time step 80 (17 in sample 2, 19 in sample 3), selected ten maximally spaced test days per subject, and trained models on pre-day windows of 30, 40, 50, 60, 70, and 80 time steps. We then simulated compliance rates from 80% down to 20% by randomly masking observations, repeating each scenario five times and averaging the outcomes. Finally, we evaluated the impact of additional EMA covariates (sleep quality, quality of life, physical activity, activity pleasantness and two social-interaction items), by comparing model forecasts on the same low-data subsets with and without these extra inputs (see Supplementary Figs. 9, 11).

### Extended methodological details for forecasting models

To ensure full reproducibility within the main manuscript, we provide here the complete structural formulations and training procedures for all forecasting models evaluated in this study.

#### Static baselines

Let *τ* be the last time point of the training data. For *t* = *τ* + 1, . . . , *τ* + 7, the last-observation carry-forward (“Last Step”) model predicted

3 $${{\bf{x}}}_{t}={{\bf{x}}}_{\tau },$$

and the global intercept model predicted the subject-specific running mean

4 $${{\bf{x}}}_{t}={\bf{\mu}}$$

with ${\bf{\mu}}:=\frac{1}{\tau}\mathop{\sum }\nolimits_{t = 1}^{\tau}{{\bf{x}}}_{t }$. A linear regression model with external inputs extended the intercept baseline via

5 $${{\bf{x}}}_{t}={\bf{C}}{{\bf{u}}}_{t-1}+{\bf{h}}+{{\mathbf{\epsilon }}}_{t},$$

where ${\bf{C}}\in {{\mathbb{R}}}^{n\times m}$ maps EMI inputs to EMA dimensions. We refer to this as a static model because any temporal variation in the predictions arises solely from time-varying inputs, not from intrinsic model dynamics.

#### Linear dynamical models

Adding a linear autoregressive component yields the VAR(1) dynamics model via

6 $${{\bf{x}}}_{t}={\bf{A}}{{\bf{x}}}_{t-1}+{\bf{C}}{{\bf{u}}}_{t-1}+{\bf{h}}+{{\mathbf{\epsilon }}}_{t},$$

with ridge-regularized coefficients chosen to ensure stability $| {\lambda }_{\max }({\bf{A}})| < 1$. The linear state-space model (Kalman filter) moves the autoregressive state to latent space **z**:

7 $${{\bf{z}}}_{t}={\bf{A}}{{\bf{z}}}_{t-1}+{\bf{C}}{{\bf{u}}}_{t-1}+{\epsilon }_{t},\qquad {{\bf{x}}}_{t}={\bf{B}}{{\bf{z}}}_{t}+{\gamma }_{t},$$

with Gaussian noise terms, and latent dimension *l* tuned by grid search. Parameters were inferred via an EM procedure with guaranteed stability using multiple random initializations to improve robustness^62^.

In this formulation, the Markovian dynamics reside in the latent transition, whereas the observation model is memoryless given **z**_*t*_. Consequently, the Jacobian used in our network analyses is determined by the latent transition (and recognition mapping), and temporal smoothing of observations affects only measurement-level correlations without changing the underlying dynamical connectivity.

#### Nonlinear dynamical models

The nonlinear SSM was implemented using the shallow PLRNN (see details in^40^). Latent dynamics obeyed

8 $${{\bf{z}}}_{t}={\bf{A}}{{\bf{z}}}_{t-1}+\Phi ({{\bf{z}}}_{t-1})+{\bf{h}}+{\bf{C}}{{\bf{u}}}_{t-1},\qquad {{\bf{x}}}_{t}={\bf{B}}{{\bf{z}}}_{t}+{{\mathbf{\epsilon }}}_{t},$$

with the piecewise-linear nonlinearity

9 $$\Phi ({\bf{z}})={{\bf{W}}}_{1}\left(\varphi ({{\bf{W}}}_{2}{\bf{z}}+{\bf{b}})-\varphi ({{\bf{W}}}_{2}{\bf{z}})\right),$$

and elementwise ReLU activation $\varphi{(\cdot)}$. PLRNNs were trained using backpropagation through time with generalized teacher forcing (GTF)^40^. Hyperparameters included latent dimension, hidden width, learning rate, batch size, sequence length, and GTF strength *α*.

#### Autoregressive transformer

The Transformer generated autoregressive predictions using an encoder-decoder architecture with multi-head self-attention, causal positional encoding, and cross-attention to EMI inputs, following the canonical Transformer design^42^. Hyperparameters included number of layers, heads, hidden size, learning rate, and sequence length. Tuning yielded different optimal sequence lengths for PLRNN (7 time steps) and Transformer (32 time steps), reflecting architectural differences: the Transformer typically benefits from longer input histories due to its self-attention mechanism, whereas the PLRNN can maintain temporal context through its internal latent dynamics and therefore performs well with shorter windows. Training used Adam.

Full parameter dimensionalities and hyperparameter search spaces are summarized in Supplementary Table 4. All models were estimated using identical computational settings.

## Supplementary information

Supplementary information

## Data Availability

The datasets generated and/or analysed during the current study are not publicly available due to shared ownership but are available from the corresponding author on reasonable request.

## Code availability

The underlying code for this study is available on GitHub and can be accessed via https://github.com/JMFechtelpeter/ema_forecast_control.
