# Supplementary material for: Computational network models for forecasting and control of mental health trajectories in digital applications
Source: NPJ Digit Med. 2025 Dec 30;9:70. doi: 10.1038/s41746-025-02252-3 (PMC12827976; doi:10.1038/s41746-025-02252-3)
Supplement: Supplementary file 1 — Supplementary information [file 41746_2025_2252_MOESM1_ESM.pdf]

Computational network models for forecasting and control of mental health trajectories in digital applications
# – Supplementary material –

November 22, 2025

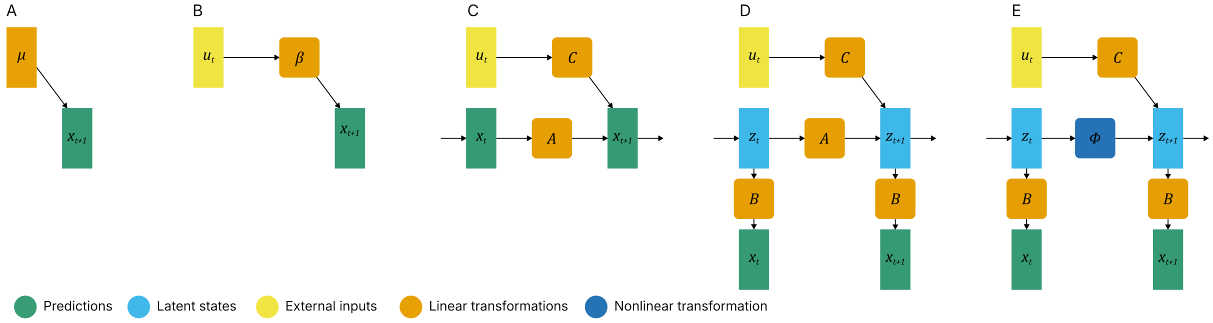

Supplementary Figure 1: **Schematic architectures of a subset of evaluated models**. A: Constant (global-mean) model. B: Adding external inputs $\mathbf{u}_t$ as predictors yields a multivariate linear regression model with coefficients β (where the offset β_0 accounts for the residual mean after modeling input effects). C: Adding a linear autoregressive term in the EMA items yields a VAR(1) model. D: Moving the recurrence to the latent space and mapping it linearly back to observations yields a linear state-space model (Kalman filter). E: Adding ReLU nonlinearity Φ to the latent space yields the PLRNN. Intercept terms and the second PLRNN layer are omitted for clarity. The "Last Step" predictor is not shown, as it can be viewed as a special (degenerate) case of an autoregressive (order 1) model (with regression coefficients = 1) and is included mainly as an important baseline benchmark. Transformer models are also omitted because their architecture does not build directly on the structures depicted here.

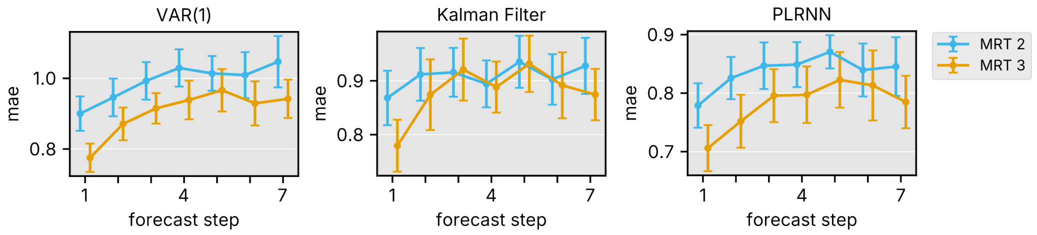

Supplementary Figure 2: **Stepwise prediction accuracy.** Stepwise mean absolute error (MAE) of predicted absolute EMA values for the VAR(1) model (left), Kalman filter (middle), and PLRNN (right). The x-axis indicates the prediction step relative to the start of the test set. Forecasting EMA values becomes progressively more difficult with time steps. Results show means and standard errors across participants.

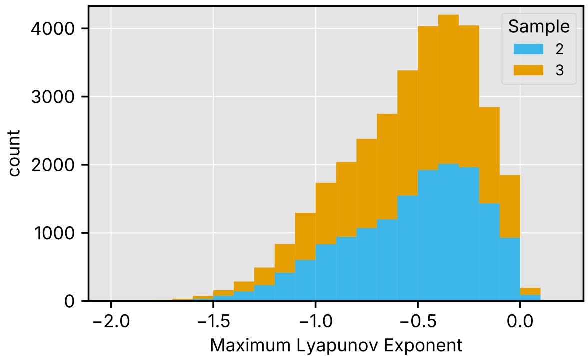

Supplementary Figure 3: **PLRNN stability analysis.** Stability of the learned nonlinear dynamics. Histogram of maximal Lyapunov exponents (MLEs) estimated for all subject-specific PLRNNs using a standard QR-based algorithm along the empirical trajectories. Negative values indicate asymptotically stable dynamics (convergence toward an attractor), values close to zero correspond to marginally stable regimes (neither con- nor divergence), and positive values to divergent or potentially chaotic behavior. Although stability in the PLRNN models was not explicitly enforced (as done in the VAR(1) and Kalman Filter models), the distribution is clearly shifted below zero, with only 0.6% of systems exhibiting an MLE above 0, indicating that the learned nonlinear models are predominantly stable in the regimes relevant for forecasting.

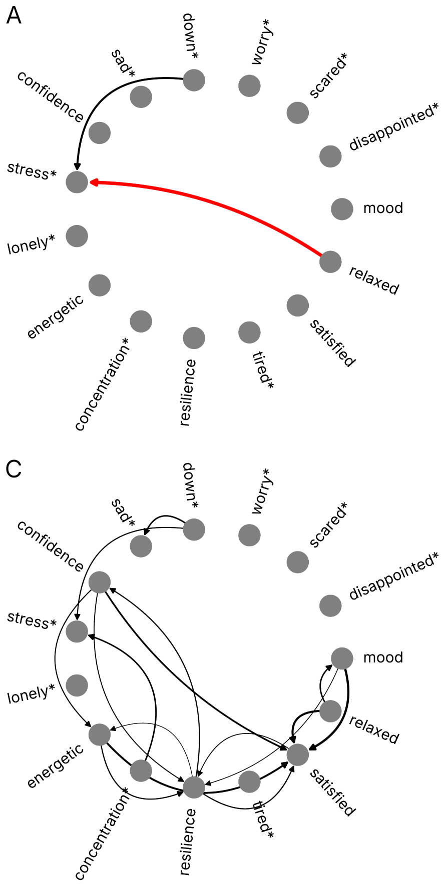

Supplementary Figure 4: **Network analysis for Kalman filter and VAR.** VAR(1) and Kalman filter models depicted as ideographic networks. Arrow width reflects mean edge strength over all participants. Positive connections are shown in black, negative connections in red (Bonferroni corrected for multiple comparisons $p_{\mathrm{Bonf}} < 0.05$). For visualization purposes, negatively poled items were not recoded and marked with an asterisk. A: VAR(1) networks from sample 2. B: VAR(1) networks from sample 3. C: Kalman filter networks from sample 2. D: Kalman filter networks from sample 3.

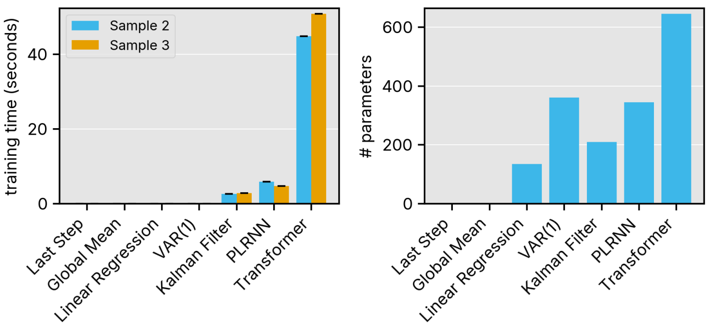

Supplementary Figure 5: **Model complexity.** Training time (left) and number of parameters (right) for each model. Results show means across participants with bootstrapped confidence intervals. The higher parameter count in PLRNN and Kalman filter models reflects their latent structure with freely scalable latent dimensions, in contrast to VAR(1), which must learn dynamics directly in the 15-dimensional observation space. Transformers also had over 1.5 times more parameters compared to PLRNNs and over 3 times more than Kalman filters in the optimal hyperparameter configuration.

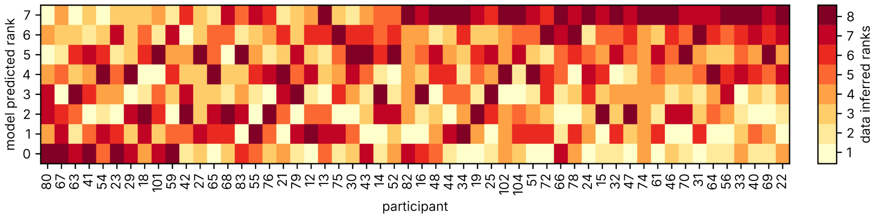

Supplementary Figure 6: **Prediction of EMI ranks for sample 2.** Empirical EMI ranks sorted by predicted EMI ranks for each participant of sample 2, ordered by weighted Spearman correlation between predicted and empirical ranks. For participants on the right side, correlations are highest (rCIRs most accurately predict proximal EMI effects). Predicted effectiveness was measured by rCIR_1, while empirical effectiveness was assessed as the one-step difference in EMA trajectories after the occurrence of an EMI. A perfect prediction would result in the order of colors shown in the right bar. This figure shows the analogue of Fig. 4a for sample 2.

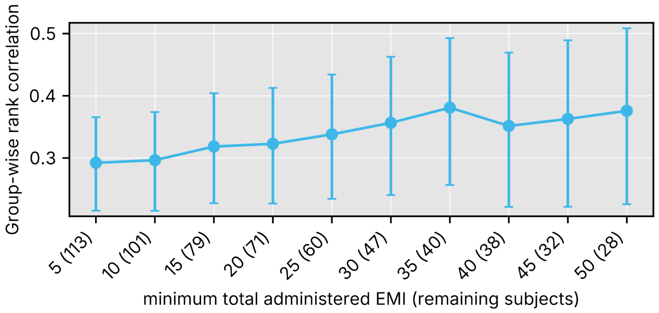

Supplementary Figure 7: **Influence of EMI count on rank predictions.** Group-wise correlation between predicted and empirical EMI effect ranks as a function of data density. At each threshold x, participants with fewer than x administered EMIs over the whole time seres were excluded, and the group-wise mean weighted correlation was recomputed. Mean and standard error are displayed. X-axis labels show the minimum number of administered EMI per participant, and in parentheses the number of subjects remaining after applying that threshold. Data from both sample 2 and 3 were included here. The visible linear trend is not significant ($F(9/599) = 0.28, p = .979$).

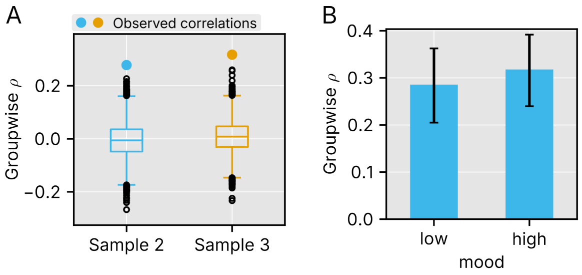

Supplementary Figure 8: **Control analyses for EMI timing biases.** A: Distribution of group-wise correlations between predicted and empirical EMI effect ranks under a null model with randomly permuted EMI timings (10,000 permutations). Boxplots summarize the permutation distributions; the blue and orange dot indicate the observed group-wise correlation using the original EMI timings. B: Group-wise correlation between predicted and empirical EMI effect ranks, computed separately for time points with mood values above versus below the median. Mood was defined as the sum over all EMA items after re-scaling negatively coded items. Mean and standard error are displayed, pooled over sample 2 and 3.

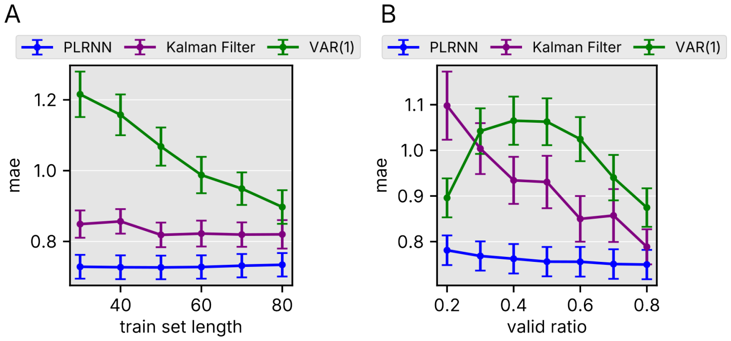

Supplementary Figure 9: **Low data limit**. A: Forecasting performance as a function of available training data. We selected participants with at least 10 eligible test days after time step 80 and >60% valid data (20 in sample 2, 17 in sample 3). For each participant, we chose 10 test days that were maximally spread out in time, and trained models on the 30, 40, 50, 60, 70, or 80 EMA steps preceding each test day, respectively. The plot shows MAE across participants (mean ± s.e.m.) as a function of training-set length. B: Forecasting performance as a function of missingness. Starting from the same participants, we simulated lower compliance rates by randomly setting time points in the training data to missing, yielding valid-data ratios of at most 80%, 70%, 60%, 50%, 40%, 30%, and 20%. This procedure was repeated five times per ratio and results were averaged; the plot shows MAE versus the ratio of valid data (mean ± standard error). Both the PLRNN and the Kalman filter exhibited reduced MAE for higher ratios of valid data, and the PLRNN achieved low prediction errors with as little as 20% valid data. The VAR(1) model performed relatively well under high levels of missingness, with its highest error around 40% valid data followed by a roughly linear decrease thereafter.

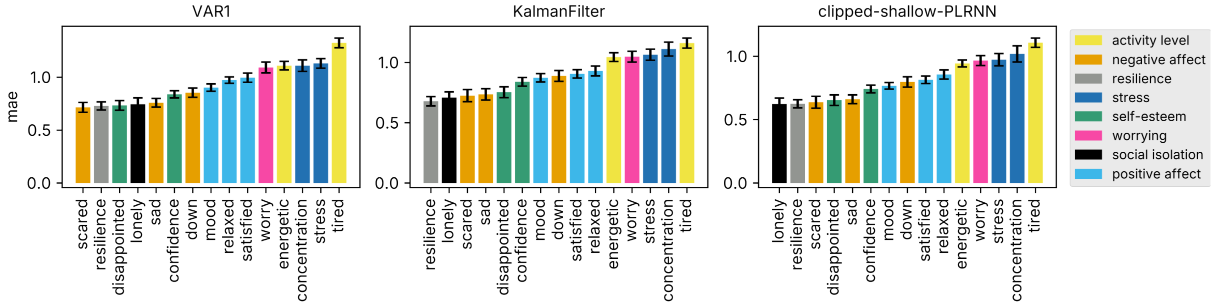

Supplementary Figure 10: **Individual item analysis.** MAE for the absolute score of each EMA item for VAR(1), Kalman filter, and PLRNN. EMA items differed substantially in their predictability as measured by MAE. Mean over participants from both samples and standard errors are displayed. The colors indicate the psychological constructs the EMA items refer to.

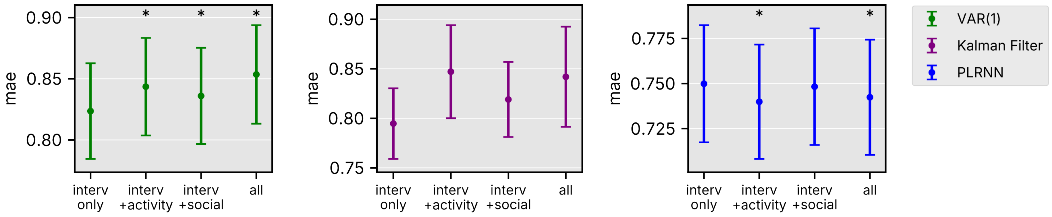

Supplementary Figure 11: **Additional covariates.** MAE for the VAR(1) model (left), Kalman filter (middle) and PLRNN (right), as a function of additional external covariates (Interv only = interventions used as external inputs, no additional covariates; interv+activity = additional activity-related EMA included as inputs; interv+social = additional social-related EMA included as external inputs; all = all additional EMA included as external inputs). All additional EMA items were normalized to the $[-1, 1]$ interval or encoded as binary variables, and appended to the input vectors $\mathbf{u}_t$; models were trained on the same subjects and prediction days as in the low-data analyses. Asterisks indicate significant differences compared to the *interv only* condition, based on paired t-tests. The PLRNN benifited significantly from including activity-related EMA items as external regressors but not social-related items, and combining both activity and social inputs also increased performance ($t(36) = 2.83, p = .008$). This analysis was performed with the same subjects as the "Low data limit" analysis, data from both sample 2 and 3 is shown here.

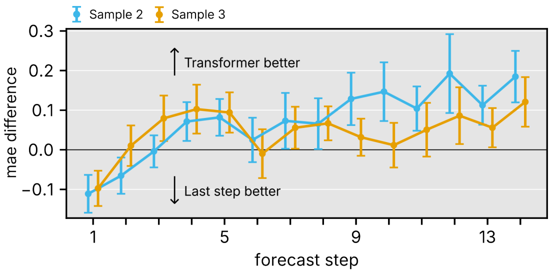

Supplementary Figure 12: **Performance of last step predictor compared to Transformer.** Per-time step difference in prediction MAE between Transformer and last step baseline for an extended prediction window of 14 steps (2 day period). Positive values indicate a lower MAE for the Transformer, negative values indicate lower MAE for the last step baseline. Overall, the last step baseline performs slightly better on the first 1-2 steps ahead and the Transformer clearly better at longer horizons.

Supplementary Table 1: **Essential data characteristics across the three AI4U samples.** For each sample, 60 participants were recruited; only those who completed the data collection phase are included here (sample 1: $n = 57$, sample 2: $n = 56$, sample 3: $n = 59$). Models were trained only on participants with at least one eligible test day (sample 1: $n = 46$, sample 2: $n = 48$, sample 3: $n = 51$). The table reports median values and 25th/75th percentiles for the number and proportion of non-missing EMA entries and the number of EMI instances. Across these key data characteristics, samples 1–3 were highly similar, and the distributions of all EMA items did not differ significantly between samples (all $p > .9$, Kolmogorov–Smirnov tests).

	sample 1	sample 2	sample 3
# subjects	57	56	59
# subjects included	46	48	51
# non-missing EMA per subject	115.5 (92.5, 175)	125.5 (102.5, 152)	127 (90, 160)
% non-missing EMA per subject	48.2 (25.4, 68.9)	49.6 (40.7, 59.9)	49.6 (35.7, 63.5)
# EMI per subject	61.5 (46.25, 88)	65 (47, 93.25)	61 (34, 87)
# of eligible days per subject	6 (3, 17.5)	7.5 (3, 14)	7 (3, 17)

Supplementary Table 2: EMA items included in this study and their properties. All were answered on a 1-7 Likert scale. Items formulated in a way that implied that high values indicated low mental well-being were reversed prior to model training and analysis.

	Code	Item	Reversed	Target construct
1	'mood'	I feel good.	no	positive affect
2	'disappointed'	I am disappointed in myself.	yes	self-esteem
3	'scared'	I feel anxious.	yes	negative affect
4	'worry'	I am pensive.	yes	worrying
5	'down'	I feel down.	yes	negative affect
6	'sad'	I feel sad.	yes	negative affect
7	'confidence'	I feel confident.	no	self-esteem
8	'stress'	I feel stressed.	yes	stress
9	'lonely'	I feel lonely.	yes	social isolation
10	'energetic'	I am full of energy.	no	activity level
11	'concentration'	I have difficulty concentrating.	yes	stress
12	'resilience'	I can handle all the difficulties I may encounter.	no	resilience
13	'tired'	I am tired.	yes	activity level
14	'satisfied'	I am satisfied.	no	positive affect
15	'relaxed'	I am relaxed.	no	positive affect

Supplementary Table 3: EMA items used as additional external inputs. Items 2 and 3 were only queried if item 1 was answered "yes", i.e. on the first EMA of the day.

	Code	Category	Item	Scale	Reversed
1	'sleep'	activity	I slept well last night.	1–7 Likert	no
2	'joyful_day'	activity	I am looking forward to the day.	1–7 Likert	no
3	'feelactive_sincebeep'	activity	I have been physically active since the last mood query.	1–7 Likert	no
4	'activity_pleas'	activity	What I am doing right now is pleasant.	-3 – +3 Likert	no
5	'social_alone_yes'	social	I am alone.	yes/no	no
6	'social_satisfied'	social	My company is pleasant / I would rather be with people.	-3 – +3 Likert	no

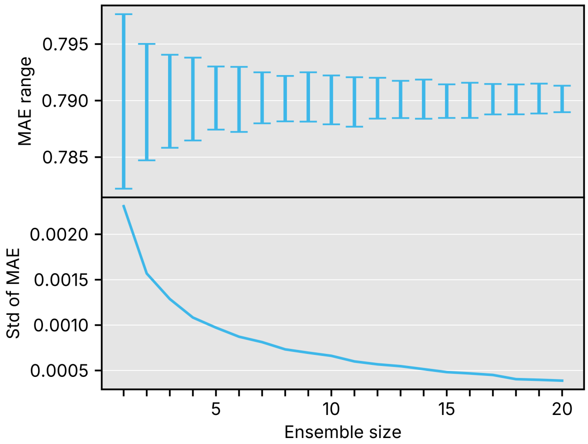

Supplementary Figure 13: **Effects of model ensemble size.** Sensitivity of ensemble size on forecasting performance. 50 models per subject were trained, and for each ensemble size we repeatedly sampled subsets from this pool of models and computed the distribution of mean absolute error (MAE) across all test days. Data from both samples were included here. Top: Range of mean absolute error (MAE) across all forecasting tasks as a function of ensemble size. Bottom: Standard deviation of the MAE as a function of ensemble size. Both the MAE range and its variability decrease markedly up to ensembles of about 8–10 models and then show diminishing returns, indicating that our choice of 10 models offers a good trade-off between computational cost and prediction stability and does not materially affect the conclusions.

Supplementary Table 4: Optimized hyperparameters and resulting total number of free parameters of all models. Optimization with MAE as target was conducted using the data from sample 1, while all subsequent model analyses were conducted using the data from sample 2 and sample 3. Hyperparameters were optimized via grid search, systematically testing all combinations of several candidate values for each hyperparameter. Only applicable hyperparameters are shown.

Hyperparameter	Linear Regr.	VAR(1)	Kalman filter	PLRNN	Transformer
latent dim			7	6	4
hidden/FC layer dim				15	5
# encoder layers					1
# decoder layers					1
# heads					1
initial learning rate				10^{-3}	10^{-3}
learning rate annealing				on	off
batch size				16	16
sequence length				7	32
TF strength α				0.125	
# free parameters	135	360	210	300	645

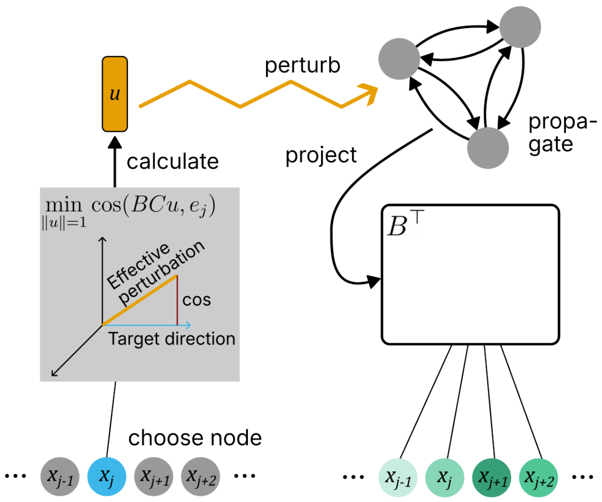

Supplementary Figure 14: **Perturbation procedure for latent models.** External inputs are added to the latent time series. To perturb a certain observation node j, we compute $\mathbf{u}$ which minimizes the cosine distance between $\mathbf{BCu}$ and the j-th unit vector, $\mathbf{e_j}$. This ensures that the perturbation, after being fed into the latent model (matrix $\mathbf{C}$) and projected into observation space (matrix $\mathbf{B}$), is targeted at item j as selectively as possible (within model constraints). To compute the CIR_T, the latent model is iterated T time steps, and the resulting predictions are summed.
